# Using tri-axial accelerometer loggers to identify spawning behaviours of large pelagic fish

**DOI:** 10.1186/s40462-021-00248-8

**Published:** 2021-05-24

**Authors:** Thomas M. Clarke, Sasha K. Whitmarsh, Jenna L. Hounslow, Adrian C. Gleiss, Nicholas L. Payne, Charlie Huveneers

**Affiliations:** 1 College of Science and Engineering, Flinders University, Adelaide, Australia; 2 Centre for Sustainable Aquatic Ecosystems, Harry Butler Institute, Murdoch University, 90 South Street, Murdoch, 6150 WA Australia; 3 College of Science, Health, Engineering and Education, Murdoch University, 90 South St., Murdoch, WA 6150 Australia; 4 School of Natural Sciences, Trinity College Dublin, Dublin, Ireland

**Keywords:** Biologging, Courtship, Kingfish, Captive, Machine learning

## Abstract

**Background:**

Tri-axial accelerometers have been used to remotely describe and identify in situ behaviours of a range of animals without requiring direct observations. Datasets collected from these accelerometers (i.e. acceleration, body position) are often large, requiring development of semi-automated analyses to classify behaviours. Marine fishes exhibit many “burst” behaviours with high amplitude accelerations that are difficult to interpret and differentiate. This has constrained the development of accurate automated techniques to identify different “burst” behaviours occurring naturally, where direct observations are not possible.

**Methods:**

We trained a random forest machine learning algorithm based on 624 h of accelerometer data from six captive yellowtail kingfish during spawning periods. We identified five distinct behaviours (swim, feed, chafe, escape, and courtship), which were used to train the model based on 58 predictive variables.

**Results:**

Overall accuracy of the model was 94%. Classification of each behavioural class was variable; F_1_ scores ranged from 0.48 (chafe) – 0.99 (swim). The model was subsequently applied to accelerometer data from eight free-ranging kingfish, and all behaviour classes described from captive fish were predicted by the model to occur, including 19 events of courtship behaviours ranging from 3 s to 108 min in duration.

**Conclusion:**

Our findings provide a novel approach of applying a supervised machine learning model on free-ranging animals, which has previously been predominantly constrained to direct observations of behaviours and not predicted from an unseen dataset. Additionally, our findings identify typically ambiguous spawning and courtship behaviours of a large pelagic fish as they naturally occur.

**Supplementary Information:**

The online version contains supplementary material available at 10.1186/s40462-021-00248-8.

## Introduction

In the past decade, the field of biologging has increasingly enabled remote monitoring of many aspects of the lives of some of the most enigmatic animals [1, 20, 49]. Devices such as tri-axial acceleration data loggers (hereafter accelerometers) allow for remote in situ assessments of animal movements and are often used in the marine realm [54, 57]. Accelerometers measure acceleration across three axes (e.g. dorso-ventral [heave], anterior-posterior [surge], and lateral [sway] axes; [54]) to generate a time-series characterising movement and activity. Accelerometers, therefore, provide an opportunity to describe and reveal behaviours of free-ranging animals based on unique characteristics of acceleration in an environment that is otherwise difficult to directly observe [10, 33, 60]. Accelerometers also provide an opportunity to measure kinematics of animals, enabling the inference of behaviours. For example, accelerometer data may be used in combination with spatio-temporal data (e.g. depth, geographic location, season) to identify ecologically-important behaviours, such as spawning [60], or feeding [6, 14, 21, 58]. Indirectly inferring occurrences of such behaviours from accelerometer data also allows for further insight into movement strategies [13, 62, 65], and energy budgets [26, 27] of animals in a free-ranging environment. Studies have used visual observations of free-ranging animals to relate acceleration profiles to individual behaviours such as different modes of travelling and feeding/drinking in Adélie penguins *Pygoscelis adeliae*, domestic cat *Felis catus* [63], and polar bears *Ursus maritimus* [41]. However, validation from time-synchronised accelerations with direct observations, referred to as ground-truthing, is required to accurately infer behaviours from accelerometer data and create labelled datasets. Other studies have identified feeding behaviours of free-ranging animals from animal-borne video, time-synchronised to accelerometer data [65, 66], but validating behaviour in situ using animal-borne cameras is limited due to cameras either being too large for small organisms, small cameras having a short battery life incapable of recording for long periods, or insufficient lighting.

To characterise fine-scale behaviours of taxa beyond binary classification (e.g. active vs inactive), studies using accelerometers are required to use high temporal resolution sampling rates ([38], but see [18]). Consequentially, datasets collected from accelerometers are exceptionally large, consisting of millions of rows of data. As a result, manually analysing accelerometer datasets is time-consuming and often one of the limiting tasks of behavioural studies using these devices [34, 54]. To overcome these limitations, more recent studies have used machine learning (ML) algorithms to train models based on patterns in the collected data allowing predictions from unseen data [3, 18, 19, 61]. Machine learning approaches address complex, large datasets that would otherwise be intractable using classical statistical techniques [28, 61]. In particular, supervised machine learners, such as random forest models, are trained on a labelled data-sets to recognise unlabelled or “unseen data” [61]. Random forest (RF) algorithms are an ensemble classifier designed to mitigate the issues associated with overfitting in decision trees through using multiple unpruned classification or regression trees [2]. These models are popular with behavioural classification data (e.g. [18, 37, 41, 62]) because they often produce higher classification accuracy than other models (e.g. k-nearest neighbour, support vector machine, naïve Bayes, adaptive boosting [2, 30, 59];). Random Forest models are able to handle thousands of mixed categorical and continuous predictions with robustness to outliers [61] and have relative ease of execution. In the case of behavioural classification of acceleration data, training an RF model to predict from unseen data means first confirming the behaviour of the animal carrying an accelerometer to match acceleration data with the corresponding behaviour class of the individual [54, 63]. Thus, a well-trained algorithm can estimate the behaviour of a free-ranging (“unseen”) individual across ecologically-important times or areas.

Understanding courtship and spawning behaviours of large marine fish is vital to predict population responses to environmental and fishing pressures, and develop suitable and adaptable management strategies [46, 51]. However, observations of spawning behaviours of most free-ranging fish is often difficult due to their patchy distribution, large-scale movements, occurrence in low light conditions, and the logistical difficulties associated with working in the marine environment). In marine fishes, accelerometers have been used to characterise a number of behavioural classes such as foraging [6], feeding [22, 58], and escape behaviours [38] based on acceleration profiles. Accelerometers have also been used to characterise complex reproductive behaviours of fishes, such as chum salmon (*Oncorhynchus keta*), flounder (*Paralichthys olivaceus*), and greater amberjack (*Seriola dumerili*) [51, 60, 71] by confirming reproductive status via either destructive gonad sampling or direct visual observations. Random forest models developed through data collected via accelerometers therefore offer an opportunity to build on this past research to address the typically challenging task of detecting natural spawning events by training a predictive model based on acceleration characteristics during visually-confirmed events, and subsequently predict naturally-occurring reproductive events on unseen data from free-ranging individuals.

Yellowtail Kingfish (*Seriola lalandi*; hereafter referred to as kingfish) is a large migratory pelagic fish, found globally in temperate and sub-tropical coastal waters [15]. In addition to being commercially and recreationally targeted, kingfish are highly palatable and consequentially part of an expanding aquaculture industry in Japan and southern Australia [44]. Access to kingfish in a captive aquaculture environment has permitted the description of their reproductive behaviours [11, 31]. Spawning most often occurs between dawn and dusk (A Miller; pers. comms, [31]) and typically involves long periods of high-speed pursuit of a female by one male, interspersed with stalling, nipping, and touching of bodies followed by the male nipping the female gonoduct, presumably to induce spawning [31]. Kingfish movement and body position during these events should, therefore, be suitably different to other behaviours, providing a unique opportunity to use accelerometers to characterise acceleration profiles of spawning behaviours [51] and develop a RF model to predict these behaviours of free-ranging kingfish..

This study aimed to describe and quantify behaviours of captive kingfish by developing a supervised ML algorithm (RF model) based on ground-truthed accelerometer data and subsequently apply this to data collected from free-ranging kingfish to identify naturally-occurring spawning behaviour.

## Methods

### Captive kingfish trials

Captive trials were undertaken at the Clean Seas Aquaculture Hatchery Facility, in Arno Bay, South Australia (33°56.222′S, 136°34.4918′E). Here, sexually mature brood stock kingfish (*Seriola lalandi*) are housed in large tanks for the purpose of ongoing production runs and egg stocking. Brood stock kingfish in production tanks are originally sourced from wild kingfish caught locally in South Australia. Between August 2018 and February 2019, two tracking sessions where undertaken where six captive brood stock kingfish (three each tracking session - 1 female and 2 males; Table 1) were tagged with tri-axial accelerometer data loggers (Technosmart Europe srl, Axy-Depth, Rome, Italy) scheduled to record at 50 Hz and +/− 2G. Loggers were programmed to record in three-axes of acceleration; surge (x), heave (y), sway (z; Figure S1) corresponding to dorsal-ventral, anterior posterior, and lateral orthogonal body axes.

**Table 1:** Description of yellowtail kingfish (*Seriola lalandi*) used for captive (C) and free-ranging (FR) accelerometer trials. Free-ranging kingfish were not checked for sex (shown as ‘-‘). Location refers to tagging location

Fish ID	Location	Date tagged	Sex	Total length (cm)	Sunrise (ACST)	Sunset (ACST)	Logger recording time (hours)
C1	Arno Bay	21/08/2018	M	91	–	–	115
C2	Arno Bay	21/08/2018	F	105	–	–	115
C3	Arno Bay	21/08/2018	M	95	–	–	115
C4	Arno Bay	8/2/2019	M	90	–	–	93
C5	Arno Bay	8/2/2019	F	97	–	–	93
C6	Arno Bay	8/2/2019	M	101	–	–	93
FR1	Neptune Islands	28/10/2015	–	99	06:28	19:51	45.5
FR2	Neptune Islands	28/10/2015	–	98	06:28	19:51	37
FR3	Neptune Islands	30/10/2015	–	114	06:25	19:53	16.7
FR4	Neptune Islands	13/2/2019	–	120	06:56	20:22	10.7
FR5	Neptune Islands	15/2/2019	–	119	06:58	20:20	16.6
FR6	Coffin Bay	10/11/2019	–	151	06:19	20:05	51.2
FR7	Coffin Bay	10/11/2019	–	142	06:19	20:05	33.7
FR8	Coffin Bay	10/11/2019	–	140	06:19	20:05	30.3

Fish were removed from holding tanks and placed inside a “knock-out tub” containing AQUI-S (10 ppm) for tagging. The logger was affixed to a padded base plate, which was attached to the fish by passing 45 kg breaking-strain monofilament leader through the dorsal musculature of the fish using sharpened embroidery needles, which had the monofilament passed through the eyelet and held secured firmly with a small crimp and heat shrink tubing. The monofilament was then cut to remove the needle and passed through a padded button (one per strand of monofilament) to act as an anchor. To identify tagged individuals, accelerometers were designed with different coloured buttons, different patterns (for night-time), as well as different numbered base plates that could be identified through video footage. Once the logger was secured, the fish was returned to the tank. A recovery period of 3 h following tagging was allowed prior to trials to allow fish to resume regular behaviours. Following trials, tagged individuals were recaptured, and loggers were manually removed.

Over 5-day periods, four video cameras (GoPro Hero 7) were placed inside the tank to enable constant recording over the trial period. Cameras were secured in waterproof housings, while running from a charging power bank battery pack to allow cameras to record for ~ 8-h periods. Time on the video recording was synchronised to the same time as the accelerometers, so that acceleration data could be directly related to the video footage for a given point in time. Trials were carried out in regular production brood stock tanks to ensure natural spawning events would occur, so there were 27 (tracking session 1) and 60 (tracking session 2) additional untagged fish within each tank over the trial periods. Fish were fed to satiation twice per day by spreading 1400–2000 g of pellet into the tank over 3–5 min. Escape behaviour was also induced during the second tracking session and consisted of a 5-min period where an extendable pole was held inside the tank with the pole moving behind tagged fish and used to instigate an escape behaviour until it was out of reach. Times of initiated feeding and induced escape behaviour were noted for synchronisation with acceleration data. During winter months (April–October) spawning in the tanks is initiated through manipulation of tank water temperatures, but during warmer months (November–March) tanks are kept at ambient seawater temperatures and spawning occurs naturally. As not all fish engage in spawning events within the tank, confirmation of courtship from tagged individuals was required and obtained through direct observation or video footage. .

### Free-ranging kingfish trials

Between October 2015 and November 2019, eight free-ranging kingfish (98–151 cm TL) were captured and tagged with accelerometers (Little Leonardo, ORI400-D3GT, Tokyo, Japan or Technosmart Europe srl, Axy-Depth, Rome, Italy) for 2–3 days (Table 1). An additional ninth kingfish was tagged with an accelerometer that prematurely released after 2 h and so was excluded from the analysis. Only fish > 80 cm total length (TL) were tagged, as this is expected to be the minimum size that both male and female kingfish are mature and to coincide with spawning behaviours [11]. Additionally, an effort was made to ensure that free-ranging fish were of similar size to the tagged captive fish to minimise any potential influence of fish size on acceleration profiles [67, 68]. Free-ranging fish (98–151 cm, mean 122.88 cm TL) in this study were, however, slightly larger than captive fish (90–105 cm, mean 96.5 cm TL). Kingfish were tagged during the Austral spring and summer months to coincided with expected natural spawning events, that are expected to be triggered by increasing water temperature and occur between spring/summer months i.e., November through April [11, 31, 43].

Accelerometers were attached to free-ranging kingfish using the same protocol as detailed above (Captive kingfish trials section) but were modified to self-detaching recoverable packages, containing an accelerometer (Axy-Depth, Technosmart Europe srl, Rome, Italy), radio transmitter (MM100, Advanced Telemetry Systems Inc., Isanti, USA), and Smart Position and Temperature transmitting tag (258, Wildlife Computers, Redmond, USA), and were deployed with corrodible links to allow for recovery after 2–3 days (Figure S1). Logger packages (138–150 g, 15 × 4 × 5.5 cm; Figure S1) were designed to be as small and streamlined as possible and of similar size to logger plates used in captive trials.

### Data analysis

Accelerometer data were downloaded and visually observed through IGOR Pro (WaveMetrics Inc., Lake Oswego, OR, USA, version 8.0.3) with add-on software Ethographer [52]. Data from periods where fish could not be directly observed or identified from the video footage due to low light conditions, or out of view of cameras were removed from further analysis.

Static acceleration (as a result of Earth’s gravitational field) and dynamic acceleration (representing body movement) were calculated for all three acceleration axes (X, Y, and Z) to filter the dominant signal caused by tail beating and body attitude, and to isolate behaviours with high amplitude acceleration (Table 2) [55, 70]. Continuous wavelet transformations were then applied to the lateral acceleration data (sway axis [Z] as a measure of tail beating undulations [47, 52];) to derive acceleration wavelets representing amplitude and stroke frequency (cycle) of tail beat cycles. The vector of the three axes of dynamic body acceleration (VeDBA) was calculated as a metric of activity level, where behaviours associated whereby increased levels of activity and metabolic rate correspond to higher values of VeDBA [4, 25, 27, 45]. Pitch and roll values were calculated based on orientation of the logger (Table 2). Absolute values for roll were used to represent the roll without influence of directionality (i.e. -90 ^o^ and + 90 ^o^ resulting in running mean average roll of 0 ^o^, or minimum value equalling 90^o^). We produced a set of descriptive predictor variables characterising kingfish behaviour for subsequent ML classification (*n* = 64, Table 2), by calculating the mean, standard deviation, skewness, kurtosis, minimum, and maximum for each of these values from 1 s increments [54] matched to known behaviour labels. Summarising acceleration data into 1-s increments was chosen as longer increments would not encompass short burst behaviours such as chafe (which only lasts 1–2 s) [7, 19, 50], in addition to it not being possible to make sub-second manual observations of behaviour.

**Table 2:** Definitions and formulae for each predictor variable measured through the accelerometer data

Variable	Formula	Definition
Static acceleration	Filtered 0.06, 0.6	1 s means for static acceleration representing body posture in each axis
Dynamic acceleration	Raw (g) – Static (g)	1 s means for dynamic acceleration representing body movement in each axis
Vector of Dynamic Body Acceleration	√Dynamic (X axis)^2^ + Dynamic (Y axis)^2^ + Dynamic (Z axis)^2^	Square root of the sum of squares of absolute dynamic body acceleration in each axis
Cycle		Cycle for the dominant frequency obtained through the continuous wavelet transformation generated spectrogram. Represents the inverse of tail-beat frequencies.
Amplitude		Amplitude for the dominant frequency obtained through the continuous wavelet transformation generated spectrogram.
Pitch	atan(X axis/(sqrt(Z axis*Z axis) + (Y axis)*(Y axis))) *180/pi	Body inclination of the fish [64] during ascending (+) or descending (−)
Roll	atan2(Z axis, Y axis)*180/Pi	Spinning movements of an individual around the main axis of the fish [53]. Absolute values for roll were used to alleviate influence of roll direction.
Standard deviation		Standard deviation of static and dynamic acceleration, VeDBA, pitch and roll in each axis.
Skewness		A measure of the symmetry of the variable
Kurtosis		A measure of the tail shape of the variable
Minimum		Minimum value of static and dynamic acceleration, VeDBA, pitch and roll in each 1 s increment
Maximum		Maximum value of static and dynamic acceleration, VeDBA, pitch and roll in each 1 s increment

Time-series acceleration data were inspected to identify potential burst-behaviours, behaviours that exceeded ±1 g acceleration in the sway axis (indicative of behaviours with high acceleration amplitude associated with tail beat), had substantial changes in roll, or periods of rapid changes in acceleration were inspected in the video footage. This conservative threshold was expected to be sufficient to detect successful spawning events based on previous descriptions of spawning in *Seriola dumerili* exceeding ±2 *g* [51]. Feed and escape behaviours were identified based on but events during times that fish were fed, or escape trials were undertaken. Behaviours were then coded (using the Ethographer mask feature) as one of five behavioural classes observed from the video: feeding, swim, escape, courtship, or chafe (Table 3).

**Table 3:** Definitions of behaviours coded from video footage that were attributed to acceleration data. Behaviours that were initiated by researchers are marked with ^a^

Coded behaviour	Definition
Feed^a^	Between 1400 and 2000 g of pellet feed was dispersed into the tank from the surface and fish were observed accelerating towards and consuming pellets. Typically lasted 3–5 min, until pellets were exhausted.
Escape^a^	Five trials per individual of 5-min in length where a researcher used a long pole to initiate burst swimming behaviour by following tagged fish with the pole until fish was out of reach. Only events where fish were visually observed to react to the presence of the pole where included as escape.
Courtship	Included both typical chase preceding spawning and actual spawning events, due to low sample size of visually confirmed spawning events (*n* = 8). A typical chase was identified from the tagged individual(s) chasing another kingfish by closely following behind or next to another fish with increase in swim speed, often rubbing nose on the underside of the body or nipping at pelvic or caudal fins [31]. Spawning was identified by a group of individuals (including tagged fish) closely rubbing bodies, followed by large burst swimming by involved individuals lasting < 10 s, and with gametes observed in the water column.
Chafe	Individual rolls to face one side of the body to the surface either in mid-water or to the bottom of the tank. Roll motion where dorsal side contacts surface or substrate in an effort to remove unwanted parasites or foreign bodies [19, 36]
Swim	Typical swim behaviours with no burst or roll events, steady lateral undulatory locomotion [19, 29]. 1500 s of swimming behaviour was allocated during periods where fish could be directly observed regularly swimming around the tank with no bursts in acceleration amplitude observed. These periods were allocated as five random, 5-min periods with good visibility, and not within 30 min of feed- or escape trials.

### Development of a machine learning classification algorithm

Random forest classification were performed using the ‘*randomForest*’ package in R (version 1.1.453). Values from each predictor variable was pooled for all captive individuals into a single dataset, before being randomly split into two datasets; 70% for training the model and 30% as a test dataset to assess the performance of the model. This partitioning of data is commonly used in machine learning applications to ensure a suitably-sized test dataset, enabling high accuracy of error estimates from acceleration data studies [37, 56]. A range of number of trees (*ntree* values) were tested starting at 500 and increasing in increments of 500 up to 2000 trees. Additionally, the number of variables randomly sampled at each split (*mtry*) were tested (in increments of 5, from 5 to 20) to assess influence on error rate of the model. It was deemed that an *ntree* value of 1000 with *mtry* default value (square root of the number of predictor variables) was sufficient based on the low range of Out-Of-Bag (OOB) error variation (5.54%), that showed minimal change when the number of trees increased beyond 1000 (Figure S2). An attempt was made to account for the unbalanced size of behaviour class data through stratified sampling with equal probabilities (function: strata, package: sampling version 2.8). However, while this stratified sampling improved precision accuracy of behaviour classes with smaller sample sized (e.g chafe), precision of courtship class was decreased and so this function was not included in the final model. The 64 predictor variables were checked for relative importance to the overall class predictive performance of the classification model with the function ‘*varImpPlot*’ within the ‘*randomForest*’ package in R, measures the mean decrease in overall accuracy if a predictor variable is removed from the model (Figure S3) [56, 61, 62].

### Evaluation of performance

Performance metrics were calculated from the RF confusion matrix as indicators of the classification performance of the model [3, 8]. A confusion matrix was created using the ‘*caret*’ package in R. This matrix provides a table of actual observations from each behaviour class (rows) versus the behaviour class predictions of the model (columns) Performance metrics were calculated from the true positive (TP), false positive (FP) and false negative (FN) observations determined by the confusion matrix [3]. True positives are observations which have successfully been assigned to the correct class by the model [3]. False Positives are observations which have been incorrectly assigned to a behaviour class. False Negatives (FN) are values that belong to a particular class, but have been incorrectly assigned to another behaviour. To evaluate performance of the model for predicting distinct behavioural classes, evaluation metrics (Pr), recall (Re), and the F-measure (F_1_) were used:

- **Recall:** Proportion of predicted behaviours from each class that were correctly classified.

$$ R= TP/ TP+ FN $$

- **Precision:** Proportion of predicted behaviours from each class that were that behaviour.

$$ P= TP/\left( TP+ FP\right) $$

- **F**_**1**_ **Score:** The harmonic mean of recall and precision. Value of 0–1, where values near 0 have low classification performance, while values closer to 1 have best classification performance

$$ {F}_1= 2 PR/\left(P+R\right) $$

### Predicting free-ranging kingfish behaviours

The RF algorithm developed using ground-truthed data from captive kingfish was subsequently applied to unseen data from eight free-ranging kingfish using the *predict.randomForest* function in R. The first 60 min of data once each fish was released was removed from the dataset, to avoid incorrect allocations of capture induced behaviours. As kingfish are physiologically robust fish [32] and anaesthetic was not used during the tagging procedure of free-ranging individuals, we assumed 60 min to be sufficient time for resuming regular behaviours. Each 1 s running mean increment of time-series data (recorded at 50 Hz) was allocated a predicted behavioural class, based on values and the same predictor variables calculated from captive fish to train the RF. To minimise misallocations of free-ranging behaviour class predictions, 1 s increments that were predicted differently to the behaviour in adjacent increments was instead allocated as the same behaviour as that which preceded it. For example, if behaviours were classified as swim, swim, feed, swim, swim; feed would be reclassified as swim. Given the improbability of behaviours occurring in a duration < 1 s, it is more likely that a predicted behaviour is the same as that which precedes that second, rather than transitioning to a new behavioural class for only 1 s. Courtship predicted from the model were categorised as either spawning events or reproductive behaviours. Spawning events were considered as each portion of the free-ranging kingfish data where courtship was predicted from the model (Figure S4). Reproductive behaviours included time periods where several spawning events were predicted over a 30–90-min period, based on descriptions from ([31]; Figure S4). Time-series data were then allocated into selected time-bins, apportioned as dawn, day, dusk, and night based on data of 1-h periods either side of sunrise (dawn) and sunset (dusk) for the location at point of capture collected from https://www.timeanddate.com (accessed 20/01/2021). Values of swimming depth (m) was also recorded once every 5 s for free-swimming fish, and values for each 1 s running-mean were allocated based on the previously filled value.

## Results

### Captive kingfish behaviour classes

**Fig. 1:**
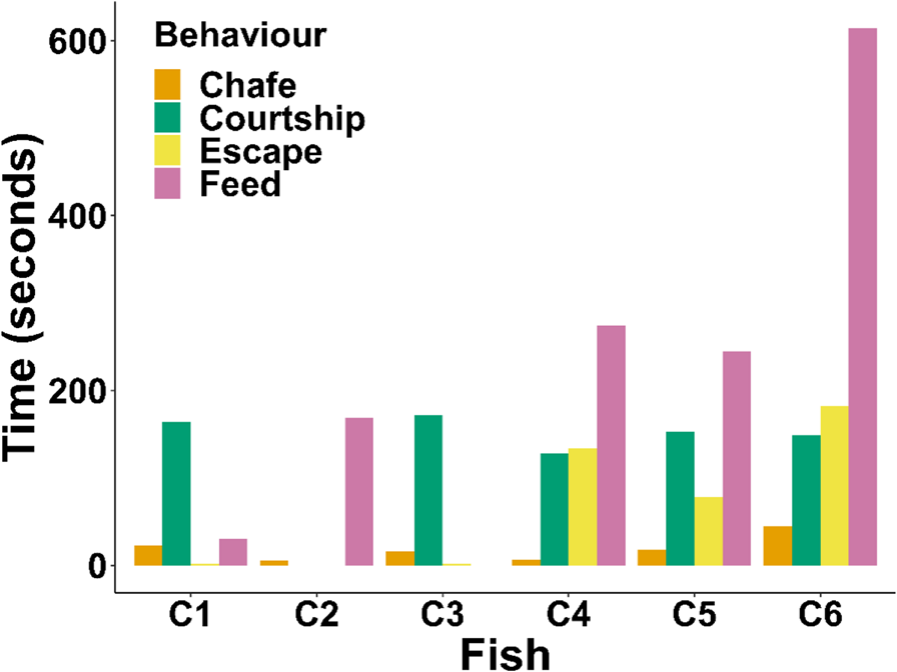
Number of 1 s increments spent performing observed behaviours for captive kingfish tagged with accelerometers, (*n* = 6, recording time = 115 h C1, 2, 3; 93 h C4, 5, 6). Swim behaviours are not included as the same amount of time swimming (1500 s) was used for each individual

A total of 624 h of acceleration data was obtained from six kingfish during captive trials over two tracking sessions at times of controlled spawning. Fertilised eggs were collected from trial tanks from 4 out of 5 and 3 out of 4 nights from Tracking session 1 and 2 respectively, confirming that successful spawning occurred on seven nights while kingfish carried accelerometers. Over 11,600 s of accelerometer data were ground-truthed and categorised as one of five behavioural classes: feed (1332 s), escape (398 s), courtship (766 s), chafe (113 s), and swim (Fig. 1). Total events (1-s increments) allocated to behavioural classes per individual varied between 1674 (C2) to 2490 (C4) seconds.

Twenty-five minutes (1500 s) of regular swim behaviour was manually coded for each captive fish (9000 s total, Table S1). Feed events were observed for all captive individuals, apart from C3. Feed classes varied between 30 and 614 s per individual, and a total of 1332 s across all fish (Fig. 1, Table S1). Courtship behaviours were observed from five fish, varying between 128 and 172 s in total per individual (Fig. 1, Table S1) and a cumulative total of 776 s. Escape behaviours were variable between individuals, with only 2 s of this class observed for C1 and C3, with up to 182 s for C6 (Fig. 1, Table S1). No escape behaviours were observed for C2. A total of 398 s of escape behaviours were collected across all fish. Chafe was the least represented behavioural class, with 113 s of this behaviour observed varying between 5 (C2) and 45 (C6) seconds in continuous length (Fig. 1, Table S1).

### Acceleration characteristics of behaviour classes

**Fig. 2:**
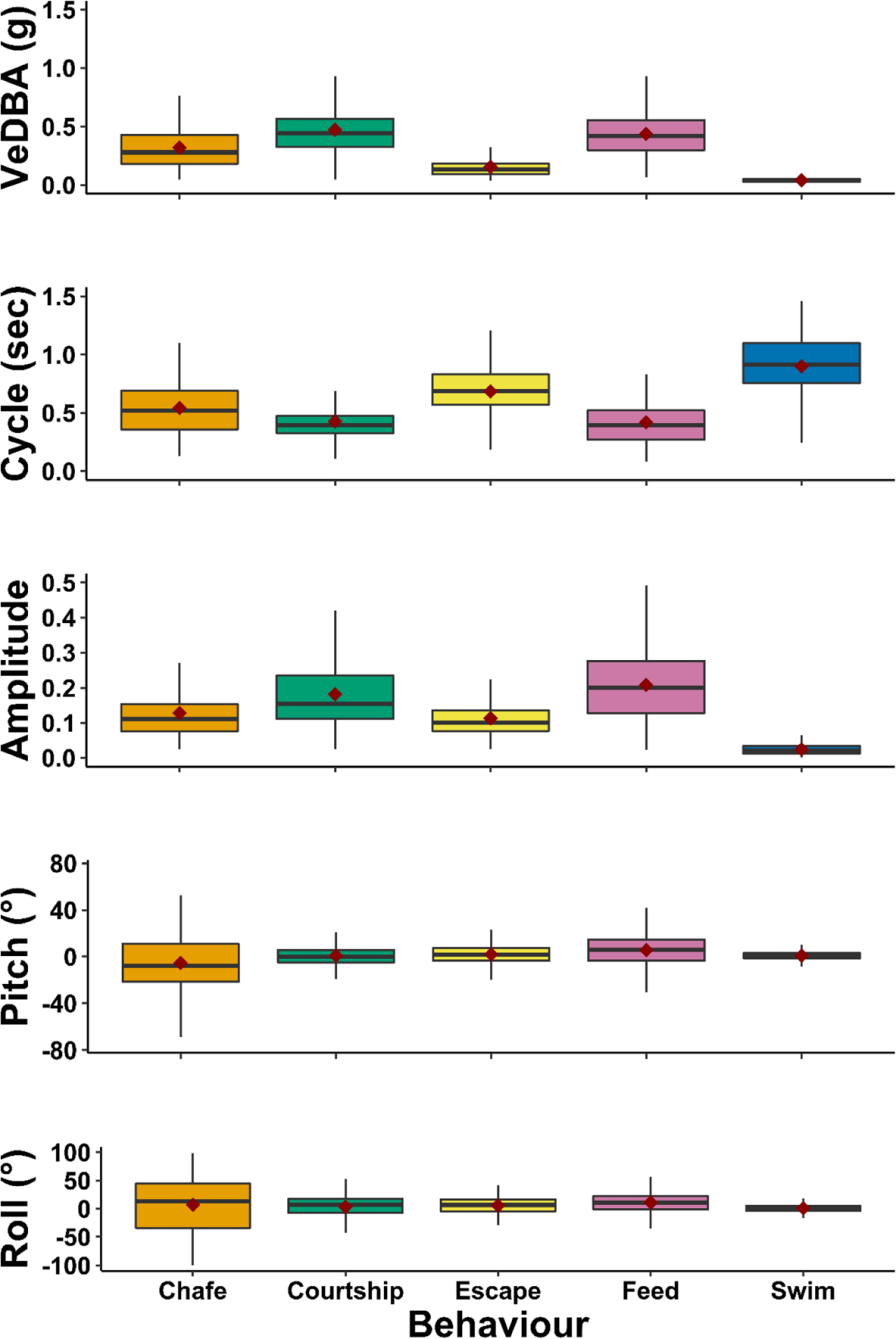
Characteristics of observed behavioural classes from captive Kingfish tagged with accelerometer loggers. Mean values shown as red diamonds. Black horizontal bars represent median values. Black boxes encompass the interquartile range, and vertical black lines represent the maximum and minimum values

Chafe behaviours were typified by a short burst (1–3 s) where fish dove to the bottom of the tank represented by a descend in depth and pitch (mean Pitch = − 6° ± 2.21), with large values of roll as fish would turn onto their sides (Fig. 2, Figure S5). Chafe included medium-strength bursts of acceleration, characterised by increased VeDBA (0.31 G ± 0.01) and tail beat (amp = 0.14 ± 0.02, cycle = 0.54 ± 0.01 s).

Courtship behaviours were typically extended periods (up to 1–2 min) of increased cycle and amplitude, interspersed with short bursts of roll and pitch (Fig. 2, Figure S5). Courtship events displayed the highest intensity acceleration of all behaviours (mean VeDBA = 0.48 ± 0.002 g), though tail beats were slightly slower and less strong than during feeding (amp = 0.19 ± 0.01, cycle = 0.43 ± 0.004 s).

Escape behaviours had acceleration signatures that were lower in intensity compared to other burst behaviours (e.g. feed, courtship) but higher than typical swimming (mean VeDBA = 0.15 ± 0.002G). Fish remained mostly upright (pitch = 2 ± 0.50 °), with intermittent small roll values (34.4 ± 0.73 °; Fig. 2, Figure S5).

Feed behaviours were identifiable by several large peaks in amplitude and VeDBA values with several large events over 3–5 min periods (Fig. 2, Figure S5). These large peaks corresponded to observed moments that fish would increase acceleration to feed on a pellet in the tank. Feed had the second highest intensity of acceleration (mean VeDBA = 0.43 ± 0.003 G), with the fastest tail beat cycles (0.42 ± 0.003 s) and highest amplitude (0.21 ± 0.006). Pitch was slightly positive (mean = 5 ± 0.38 °) due to fish ascending towards the surface to consume pellets.

Swim behaviours were characterised by steady, low values of acceleration, pitch, roll, and cycle, and consistent low peaks of cycle demonstrative of the regular occurring tail beat of the individual (Fig. 2, Figure S5). During swim, VeDBA was lowest out of all classes (mean VeDBA = 0.04 ± 0.44 G), which was due to slow, long-duration tail beats (amp = 0.02 ± 0.006, cycle = 0.9 ± 0.003 s). During swim, body position of fish remained upright, resulting in roll values close to zero (mean roll = 0.34 ± 0.7).

### Model classification performance of behavioural classes

Overall model accuracy of the RF model was 94%. The RF model predicted behaviour classes with variable performance (F_1_ ranging from 0.46–0.99). Classification performance (F_1_) was highest for swim and feed classes (> 84% accuracy, Table 4), followed by courtship, with lower allocation scores for escape and chafe classes (Table 4).

**Table 4:**
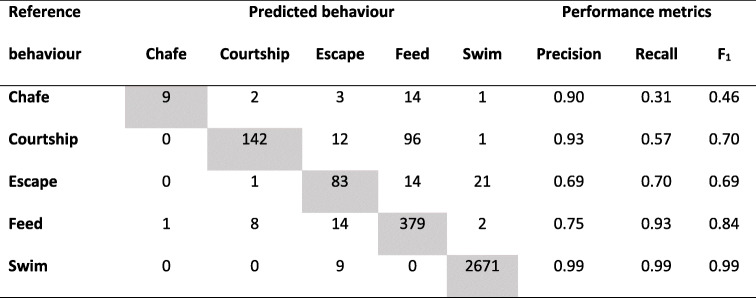
Performance metrics of behavioural classes from captive kingfish calculated from random forest algorithm on the test data (30% overall). Grey boxes represent number of correctly allocated behaviour increments from test data set

Swim class had the highest correct allocation of all behavioural classes, with all performance metrics exceeding 0.99 (Table 4). Swim was correctly predicted for all 1 s increments, except for 9 s which were incorrectly allocated as escape. Feed was the next highest performing behavioural class. Somewhat low precision for the feed class (0.75) caused by feeding behaviours being predicted by courtship, chafe, and escape, was complemented by high recall (0.93), resulting in an F_1_ score of 0.84. Courtship had a high level of correctly allocated predictions (precision = 0.93), on only 11 occasions was incorrectly allocated as feed, chafe, or escape. Courtship had a lower score for recall (0.57) as a result of misallocations of actual courtship events being predicted incorrectly, predominantly as feed. This resulted in an F_1_ score for the courtship class of 0.70. Escape was largely correctly allocated (precision = 0.69), though 17 and 12% of escape behaviours were incorrectly allocated as swim and feed classes, respectively. The model incorrectly predicted courtship (9%) and feed (12%) occurrences as escape resulting in a recall score of 0.70, and an overall F_1_ of 0.69. Chafe was the poorest performing behavioural class, with only 31% of chafe behaviours correctly allocated as this class. Most of these behaviours (48%) were incorrectly predicted as the feeding class. Although chafe had a high result for precision (0.90) representative of the model only incorrectly predicting chafe to occur once, as feeding. These values resulted in a F_1_ score of 0.46.

### Free-ranging kingfish behaviours

**Fig. 3:**
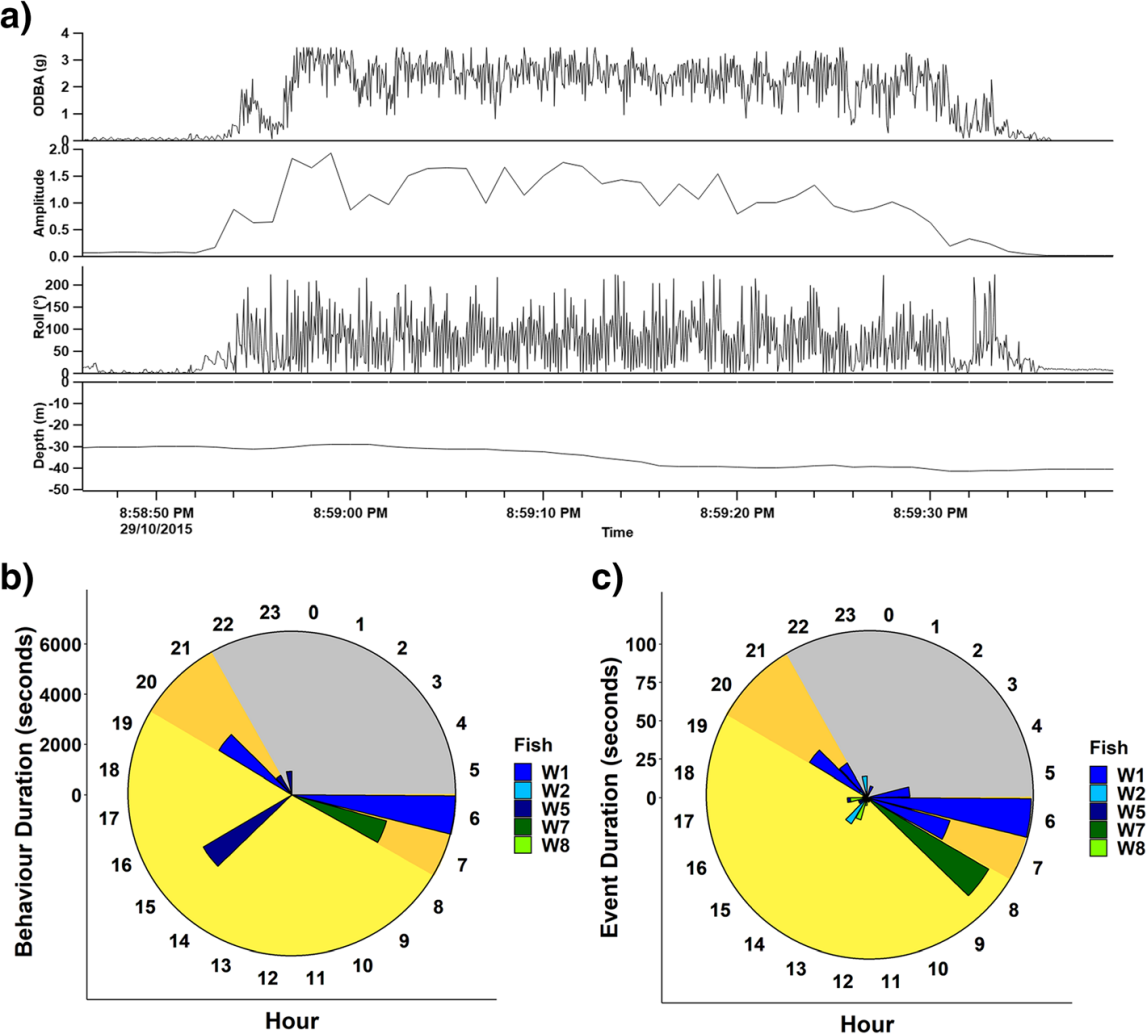
Example of one free-ranging kingfish reproductive event predicted from the random forest model (**a**), and, total duration in seconds of (**b**) reproductive behaviours and (**c)** spawning events predicted from free-ranging yellowtail kingfish individuals at the Neptune Islands (blue) and Coffin Bay (green) as predicted from a supervised machine learning model. Different colour shades represent an individual fish. Time of day is indicated by dawn (orange), dusk (orange), day (yellow) and night (grey)

The model trained using the entire ground-truthed dataset was used to predict naturally-occurring behaviours from eight free-ranging kingfish (Fig. 3). All five behavioural classes observed and coded in the captive trials were predicted to be performed by free-ranging individuals. Five of the eight free-ranging fish spent most of the recording period swimming (67–97%; Figure S6), however the remaining three fish (FR4, FR5 and FR7) mostly displayed escape behaviour, accounting for 81, 58, and 45% of behaviour class allocations, respectively.

Courtship was predicted to occur from five free-ranging fish, three from the Neptune Islands and two from Coffin Bay. A total of 48 spawning events were predicted and classified into 19 expected reproductive behaviours. Reproductive behaviours were typically made up of between 1 to 6 spawning events, with one reproductive event containing 18 spawning events (Figure S7), lasting 108.6 min (Fig. 3b). Of the 19 events, 12 included only single spawning events (Figure S7). Of these single occurrence events, duration ranged from 3 to 14 s (Fig. 3c).

Reproductive behaviours occurred between 12 and 103 m of depth at the Neptune Islands (*n* = 16 events, mean = 35.8 ± 3.84 m) and 1–6 m in depth at Coffin Bay (*n* = 5 events, mean = 2.98 ± 0.42 m). During courtship events, fish exhibited minimal changes in depth, generally propelling slightly towards the surface 0.74 ± 0.18 m in Coffin Bay, 1.81 ± 0.38 m at the Neptune Islands (Fig. 3a). Courtship predominantly occurred at dawn and day, with 17 and 18 events (accounting for 35 and 38% of all spawning events respectively. Comparatively, 7 (15%) and 6 (13%) of the predicted events occurred at night and dusk (Fig. 3c).

## Discussion

This study used accelerometers and machine learning to detect and describe reproductive behaviours of yellowtail kingfish in a captive environment, differentiate these from other behaviours, i.e. swimming, feeding, escape behaviours, and to identify these behaviours in free-ranging kingfish. Our results showed that all behaviour classes were predicted from free-ranging kingfish, with swimming and escape behaviours being most common. Evidence of courtship was observed from five free-ranging kingfish, supporting the occurrence of reproductive behaviour and events at both the Neptune Islands and Coffin Bay, and further encouraging the combined use of accelerometers and machine learning as a tool to identify naturally-occurring behaviours of large pelagic fish.

Accelerometers provide an opportunity to record in situ movements of free-ranging organisms to infer behaviours based on changes in acceleration, body position, and tail beat signatures [23, 69]. Previous applications of this method to verify ecologically-important behaviours of large pelagic fishes have been limited to identifying and describing spawning events of marine fishes based on visualisation of tail beat acceleration signatures [51, 71]. However, accelerometers have not yet been used to distinguish spawning from other naturally-occurring burst behaviours. We successfully identified and described five behavioural classes of yellowtail kingfish, including courtship, based on variables characterising acceleration profiles, body position, and tail beat signatures. While visual observation of data obtained from accelerometer loggers was sufficient to identify burst behaviours (e.g. courtship, feed, escape), the random forest algorithm was necessary to differentiate among burst behaviours.

Swimming behaviour in pelagic fish is represented by regular, low intensity cyclic patterns in sway acceleration which is more regular and consistent compared to infrequent, high intensity burst behaviours [4, 12]. These predictable waveform signals are typical in pelagic fish and sharks [12, 19], contributing to forward propulsion and is commonly the most highly allocated behaviour in machine learning studies differentiating behaviours of swimming marine organisms given that these behaviours are the most frequently performed behavioural class [19]. These expectations were met by our model, with consistent high levels of successful allocations for swimming behaviour. However, differentiating behavioural classes with more complex kinematics of movement, and high and variable frequency and amplitude such as burst behaviours, has been a challenge for most accelerometer-based studies of behavioural predictions [6, 51].

While courtship and spawning were visually confirmed from five of the six tagged captive kingfish, low light levels when courtship and spawning mostly occurred, i.e. at night, dusk, and dawn [31, 43], limited the number of spawning events which could be visually confirmed via video footage. Induced spawning through hormone injections could have been used to increase the number of spawning events observed (e.g. [51]), but the acceleration signature from such events may not be representative of kinematics exhibited during naturally-occurring spawning (i.e. less courting or chasing), limiting its use to infer spawning events in free-ranging fish. Likewise, the use of artificial lighting to improve visibility in low light conditions (e.g. at night) could affect fish behaviour and prevent spawning from occurring [5, 17, 24]. Instead, increased observation effort during dawn and dusk when spawning is expected to occur would likely increase the number of reproductive behaviours observed and improve predictive capacity of naturally-occurring courtship in the future.

The most frequently predicted behaviour of free-ranging kingfish at both the Neptune Islands Group and Coffin Bay was swimming, providing some support for the predictive power of our approach given it would be expected that swimming should be occurring a majority of time by free-ranging individuals. However, escape was the most frequently predicted behaviour in three free-ranging fish, but it is unlikely that these fish spent more time escaping predators than swimming across 1–2 days. Acceleration signatures are also influenced by body size and locomotion of pelagic schooling fish [40], which may result in misclassification of behavioural classes between differently sized individuals. In addition to body size, free-ranging kingfish may exhibit larger tail strokes than captive fish constrained in tanks, resulting in fast regular swimming in free-ranging fish being allocated to escape behaviours. While we attempted to minimise these effects by attaching loggers to fish of similar size in both captive and free-ranging environments, free-ranging fish were slightly larger than captive individuals. Escape behaviours invoked by pursuing individuals with a pole in the captive environment may also not be sufficiently comparable to natural escape responses in a free-ranging environment. For example, kinematics exhibited while escaping predators would likely differ from manoeuvres performed in tanks. Therefore, this may lead to the escape behaviours from the captive fish poorly translating in the model, contributing to high allocations of escape in some free-ranging individuals. Although this approach is effective for identifying some behaviours (e.g. courtship, feeding), the constraints of validating naturally-occurring behaviours in tanks can affect the accuracy of detecting others (i.e. escape) that are not well translated from captive to natural environments.

Access to captive kingfish from the aquaculture industry has previously enabled research describing spawning and reproduction in captivity [31, 43]. Our study reveals, for the first time, information about the timing and locations of kingfish spawning in the wild. A total of 19 reproductive behaviours were identified in five of the eight free-ranging fish, although Thirteen behaviours lasted less than 1 min and might be misallocated behaviours. The six remaining behaviours lasted 14–109 min which is of similar duration to reproductive behaviours described by [31]. All but two of these behaviours occurred at dawn or dusk, resembling theories of spawning occurring predominantly during low light levels, e.g. dawn [31, 43] and supporting the validity of our results here. Courtship was identified from fish tagged at both study sites, the Neptune Islands and Coffin Bay. While it is possible that the kingfish left the vicinity of the Neptune Islands or Coffin Bay after tagging, the location of the loggers upon recovery and high residency of kingfish at both these locations (T. Clarke, unpublished data) suggest that the spawning observed occurred in areas within Coffin Bay and around the Neptune Islands. While these findings of courtship are encouraging, predicted courtship events which do not match the described spawning profiles of kingfish may have been misclassified from other burst event behaviours with similar acceleration profiles. This reiterates that behaviours identified by the RF model should be carefully reviewed and ground-truthed where possible regardless of the high accuracy and performance of the model [61]. It is also possible that the identified courtship events did not result in the release of gametes, which affects the suitability of using accelerometers to infer spawning events [16]. However, even if the tagged fish does not release gametes, the indication that courtship or spawning attempts are taking place is a reliable indication of timing and areas used for reproductive events [16] and that accelerometers can help identify when spawning is occurring. Integrating additional sensor (e.g. depth sensor) or tags (e.g. acoustic or satellite tags) means we can also use accelerometers to infer patterns in migrations, as well as spatial and habitat use to determine spawning location and depth for spatial and temporal management efforts to protect spawning aggregations [9, 42, 48].

A limitation of behavioural studies such as ours using accelerometers is the individual variation in behaviours, movements, and swimming performance between captive and wild individuals. Captive environments are space-limited and often have manipulated conditions which may skew its performance for use on datasets from free-ranging individuals. In addition, ML algorithms such as the RF model developed in this study do not account for the temporal auto-correlation expected in timeseries [3, 28]. While there are very few ways to combat these issues (e.g. Hidden Markov models [28];), future studies may attempt to ground-truth data in the wild environment through means of animal-born video cameras. However, these technologies come with a trade-off of limited battery-life, low visibility under poor light conditions, and additional weight to accelerometer packages potentially influencing behavioural profiles.

## Conclusions

Combined use of accelerometers and supervised machine learning algorithms has become prevalent as a method of characterising behaviour classes from both terrestrial and marine taxa. Our study builds on past work, which has been predominantly constrained to direct observations of behaviours, by applying such models on free-ranging data in a natural environment. Through direct observations of courtship and spawning behaviours, our findings provide the first study to predict naturally-occurring courtship of a large pelagic fish, yellowtail kingfish, via the use of accelerometers and ML. These findings contribute to more detailed approaches of identifying naturally-occurring behaviours, which in the past have been only inferred through increase in general activity patterns, or destructive sampling approaches. This method may contribute to a detailed understanding of timing and location of important reproductive aggregations of large pelagic fish, and in turn the effective spatial and temporal management strategies required to protect spawning populations.

## Supplementary Information

**Additional file 1: Figure S1.** Orientation of accelerometer packages attached to a) captive, and b) free-ranging Yellowtail Kingfish. Loggers remained attached for 2–3 days after which a corrodible link releases the tag to the surface for collection. **Figure S2.** Error rate of Random Forest model with increasing number of trees (ntree). **Figure S3.** Variable importance plots for predictor variables Mean decrease in accuracy shows how model performance decreases if a predictor variable is removed from the model, and mean decrease in Gini Index shows the importance of a predictor variable based on Gini Impurity Index for the calculation of splits in trees. **Figure S4.** Example of spawning events (*n* = 16, pink markers) and reproductive behaviours (*n* = 1, orange arrow) as predicted from RF model applied on free-ranging Kingfish. **Figure S5.** Acceleration signatures for A) swim, B) escape, C) chafe, D) feed, and E) courtship behavioural classes from captive Yellowtail Kingfish recorded via accelerometer loggers. **Figure S6.** Number of 1 s increments predicted from free-ranging Yellowtail Kingfish at each hour of the day. Time of day is indicated by dawn (orange), dusk (orange), day (yellow) and night (grey). **Figure S7.** Number of a) reproductive behaviours and b) spawning events predicted from free-ranging Kingfish at the Neptune Islands (blue) and Coffin Bay (green), as predicted from the Random Forest model. Time of day is indicated by dawn (orange), dusk (orange), day (yellow) and night (grey). **Table S1.** Total number of seconds for each behaviour class from captive Yellowtail Kingfish used to train the Random Forest model.

## Data Availability

Data and material including acceleration data, video recordings, tagging information is available upon request from the authors. We recognise no competing interests.
