# Supplementary material for: Using tri-axial accelerometer loggers to identify spawning behaviours of large pelagic fish
Source: Mov Ecol. 2021 May 24;9:26. doi: 10.1186/s40462-021-00248-8 (PMC8145823; doi:10.1186/s40462-021-00248-8)
Supplement: Supplementary file 1 — Additional file 1: Figure S1. Orientation of accelerometer packages attached to a) captive, and b) free-ranging Yellowtail Kingfish. Loggers remained attached for 2–3 days after which a corrodible link releases the tag to the surface for collection. Figure S2. Error rate of Random Forest model with increasing number of trees (ntree). Figure S3. Variable importance plots for predictor variables Mean decrease in accuracy shows how model performance decreases if a predictor variable is removed from the model, and mean decrease in Gini Index shows the importance of a predictor variable based on Gini Impurity Index for the calculation of splits in trees. Figure S4. Example of spawning events (n = 16, pink markers) and reproductive behaviours (n = 1, orange arrow) as predicted from RF model applied on free-ranging Kingfish. Figure S5. Acceleration signatures for A) swim, B) escape, C) chafe, D) feed, and E) courtship behavioural classes from captive Yellowtail Kingfish recorded via accelerometer loggers. Figure S6. Number of 1 s increments predicted from free-ranging Yellowtail Kingfish at each hour of the day. Time of day is indicated by dawn (orange), dusk (orange), day (yellow) and night (grey). Figure S7. Number of a) reproductive behaviours and b) spawning events predicted from free-ranging Kingfish at the Neptune Islands (blue) and Coffin Bay (green), as predicted from the Random Forest model. Time of day is indicated by dawn (orange), dusk (orange), day (yellow) and night (grey). Table S1. Total number of seconds for each behaviour class from captive Yellowtail Kingfish used to train the Random Forest model. [file 40462_2021_248_MOESM1_ESM.docx]

**Supplementary Material: Using tri-axial accelerometer loggers to identify spawning behaviours of large pelagic fish**

^1^Thomas M. Clarke, ^1^Sasha K. Whitmarsh, ^2^Jenna L. Hounslow, ^2^Adrian C. Gleiss, ^3^Nicholas L. Payne, ^1^Charlie Huveneers

1 College of Science and Engineering, Flinders University, Adelaide, Australia

2 Centre for Sustainable Aquatic Ecosystems, Harry Butler Institute, Murdoch University, Perth, Australia

3 School of Natural Sciences, Trinity College Dublin, Dublin, Ireland


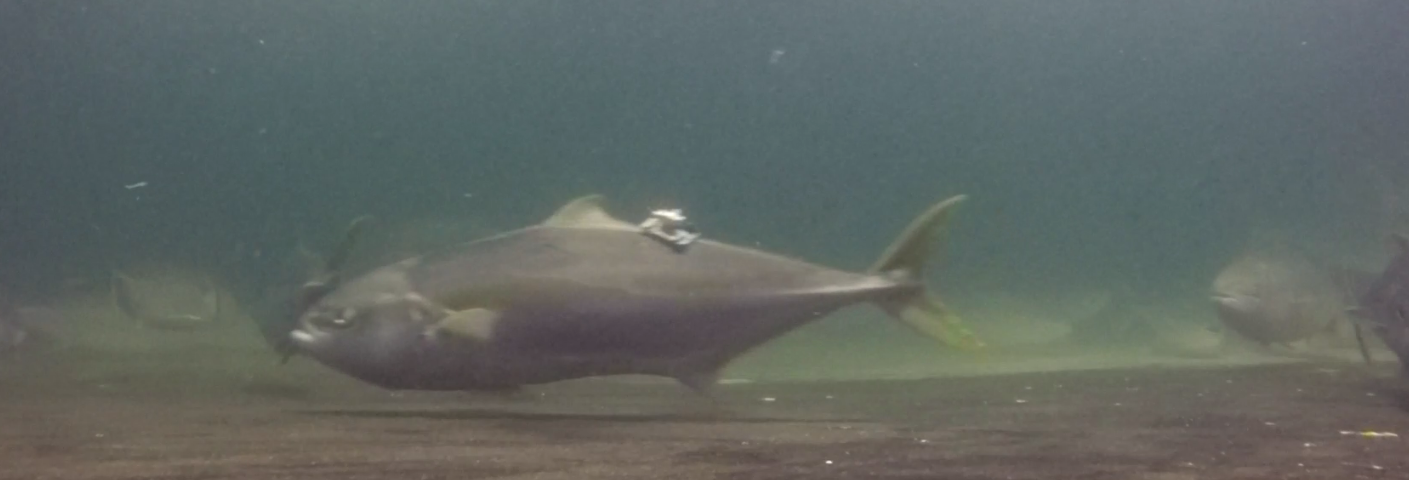


**Surge (x)**

**Heave (y)**

**Sway (z)**

a)


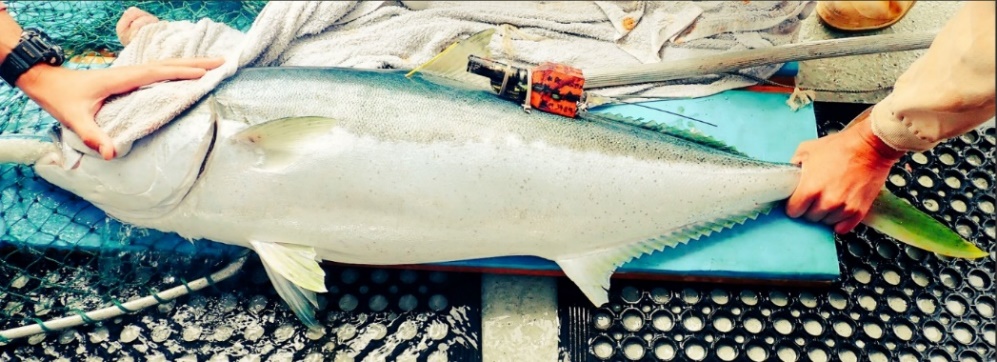


b)


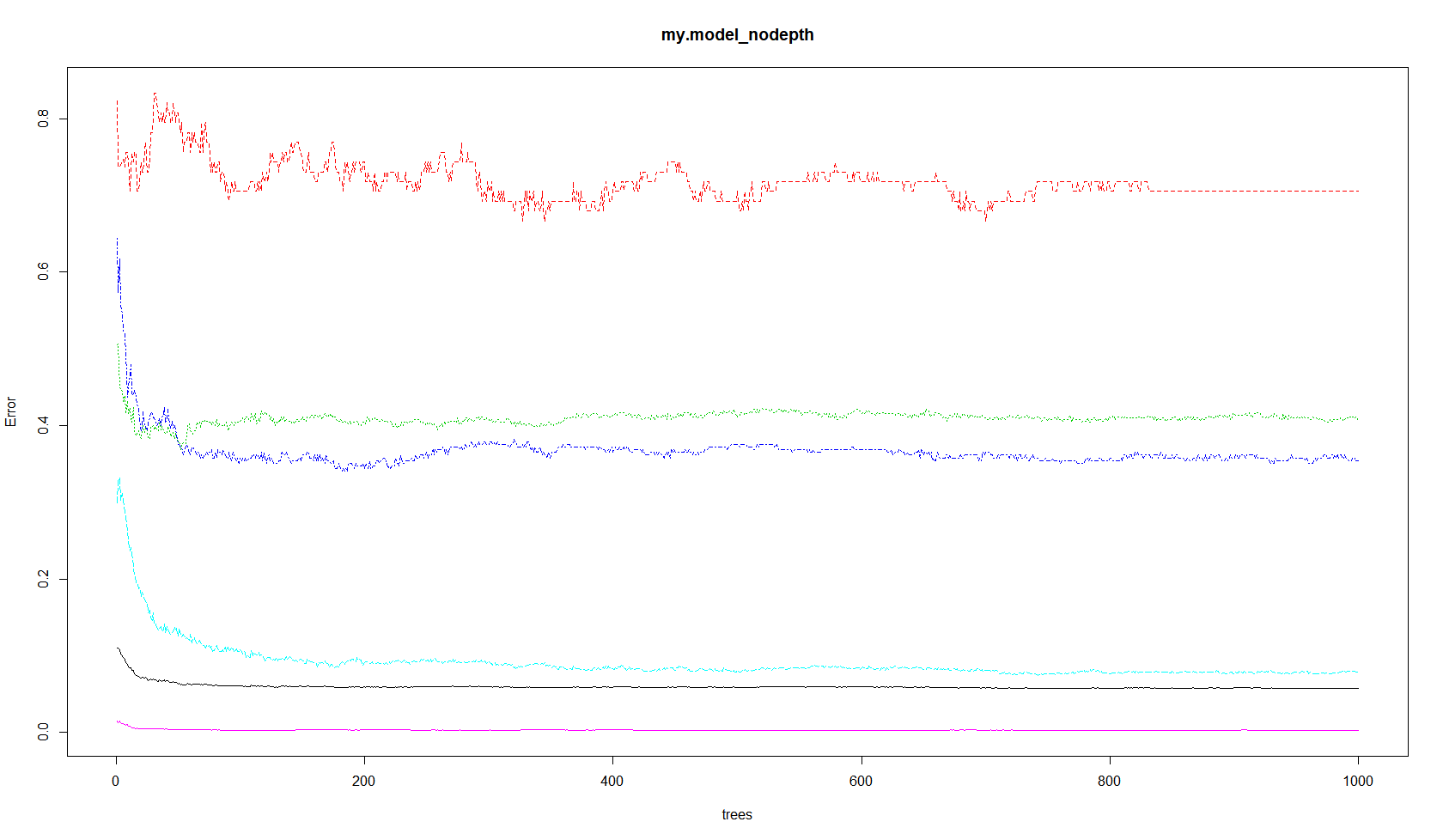
***Figure S1.*** *Orientation of accelerometer packages attached to a) captive, and b)free-ranging Yellowtail Kingfish. Loggers remained attached for 2 – 3 days after which a corrodible link releases the tag to the surface for collection.*

***Figure S2.*** *Error rate of Random Forest model with increasing number of trees (ntree)*

***
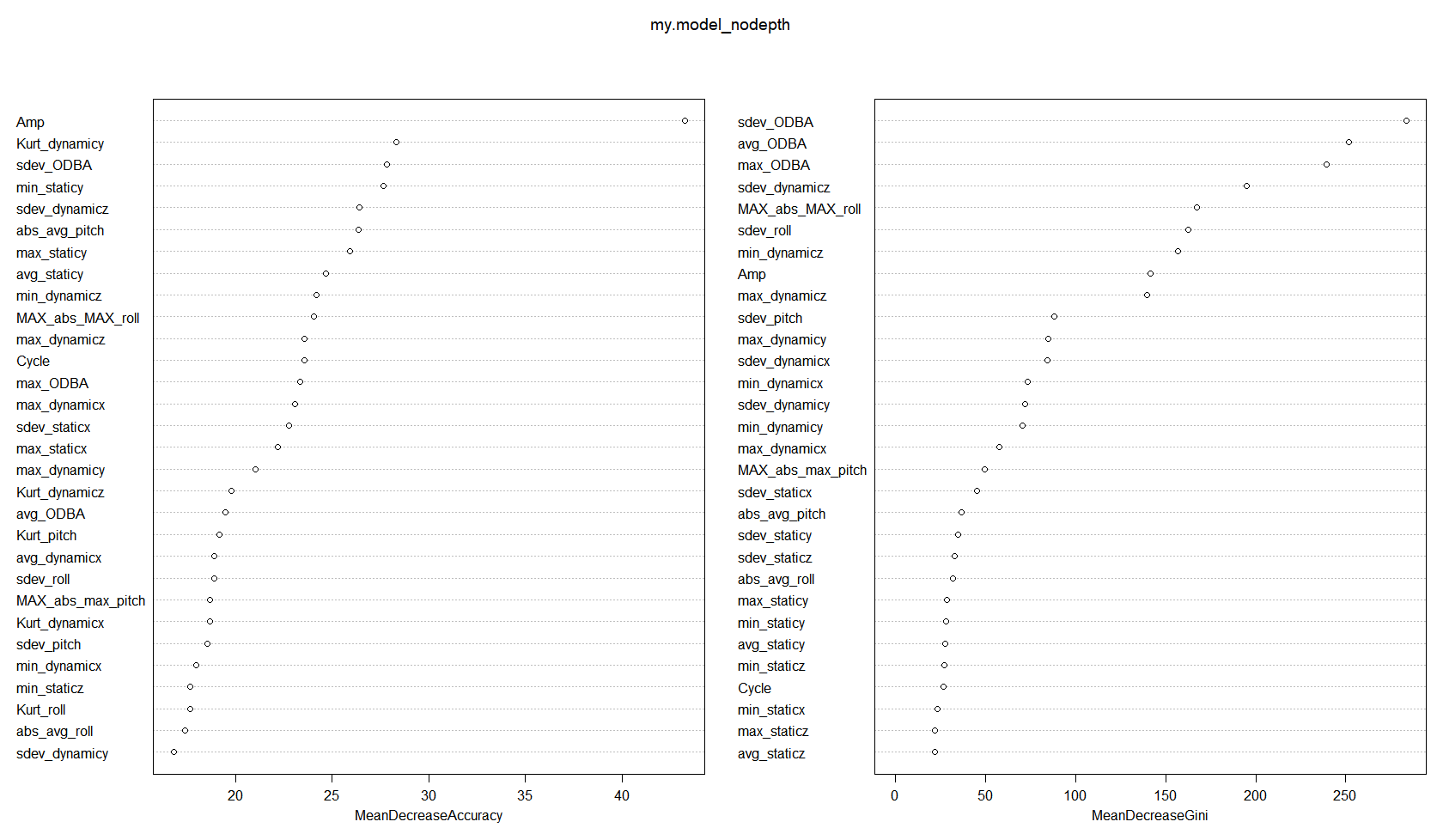
Figure S3.*** *Variable importance plots for predictor variables Mean decrease in accuracy shows how model performance decreases if a predictor variable is removed from the model, and mean decrease in Gini Index shows the importance of a predictor variable based on Gini Impurity Index for the calculation of splits in trees.*

**
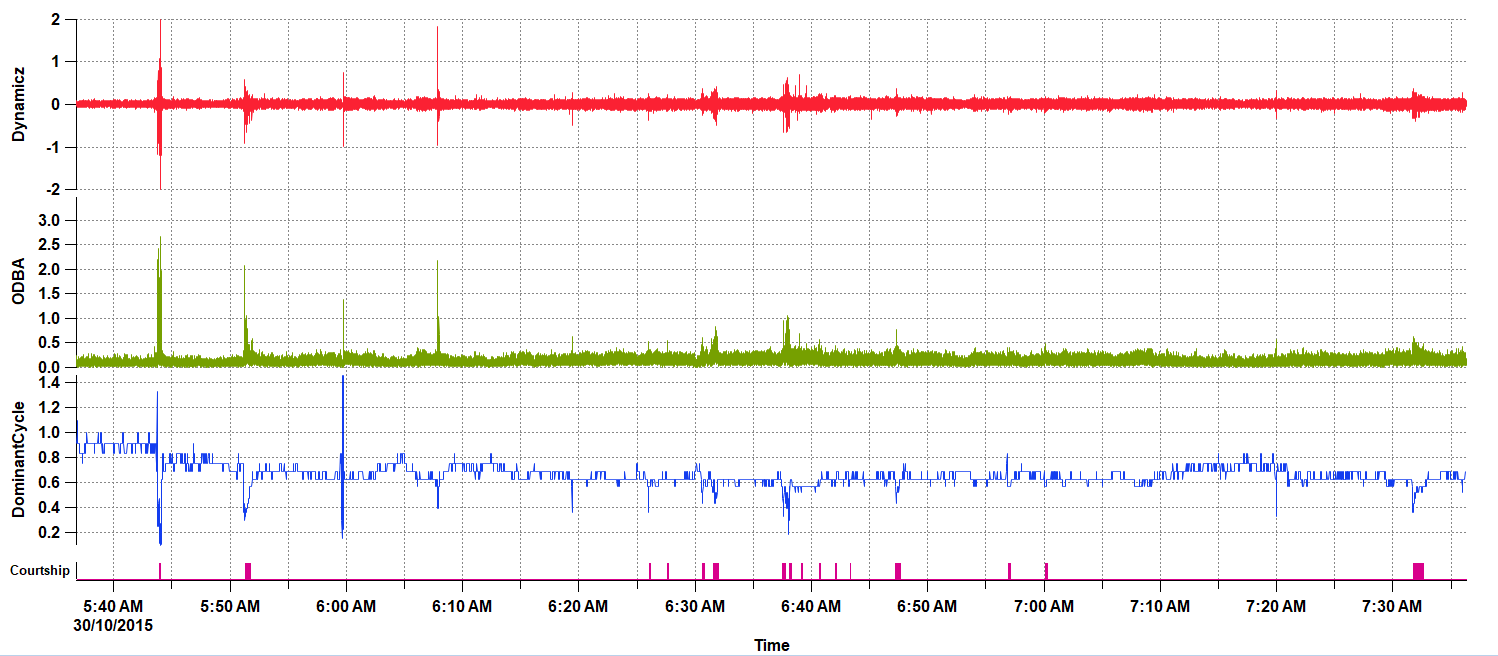
**

***Figure S4****. Example of spawning events (n = 16, pink markers) and reproductive behaviours (n = 1, orange arrow) as predicted from RF model applied on free-ranging Kingfish*


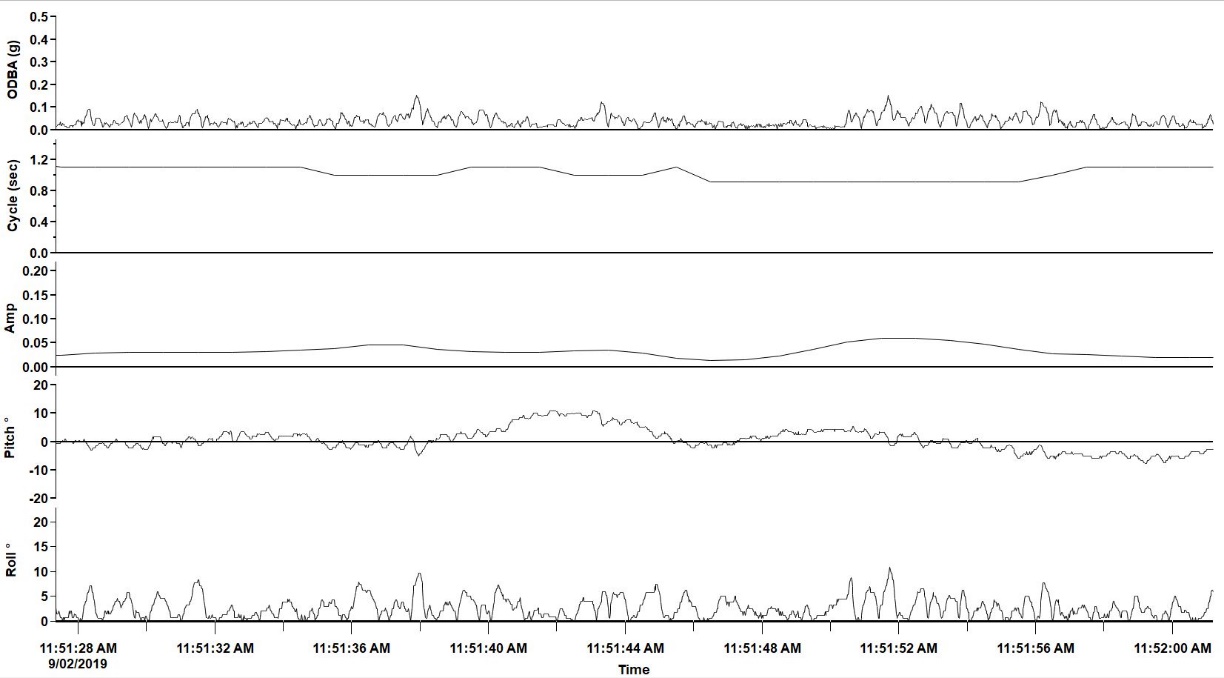


**A)**


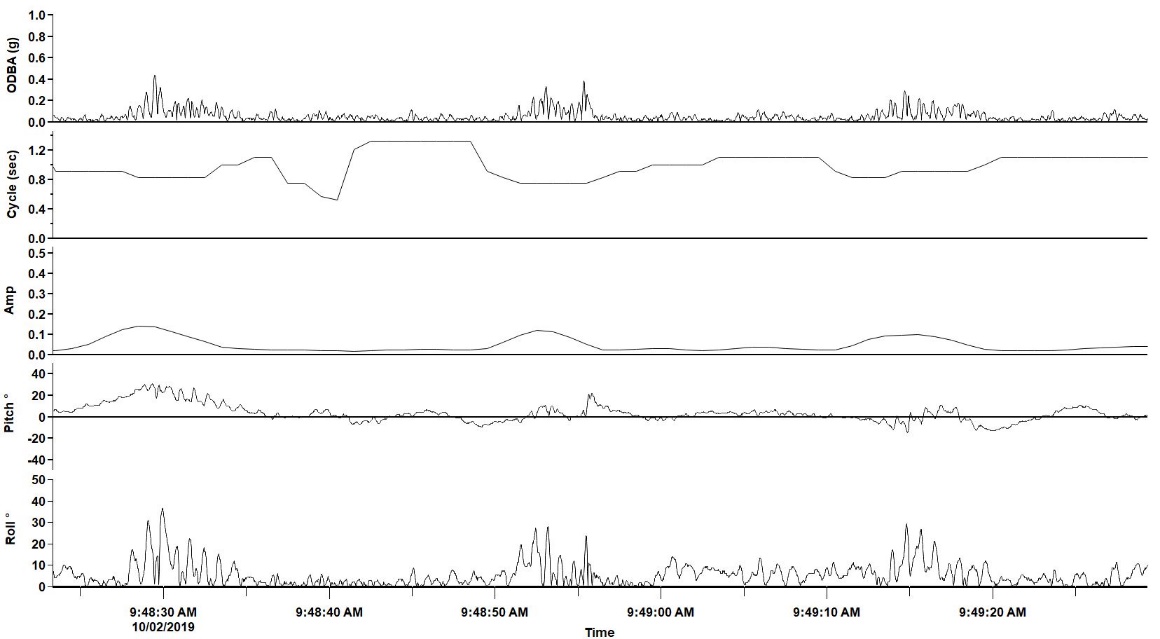


**B)**


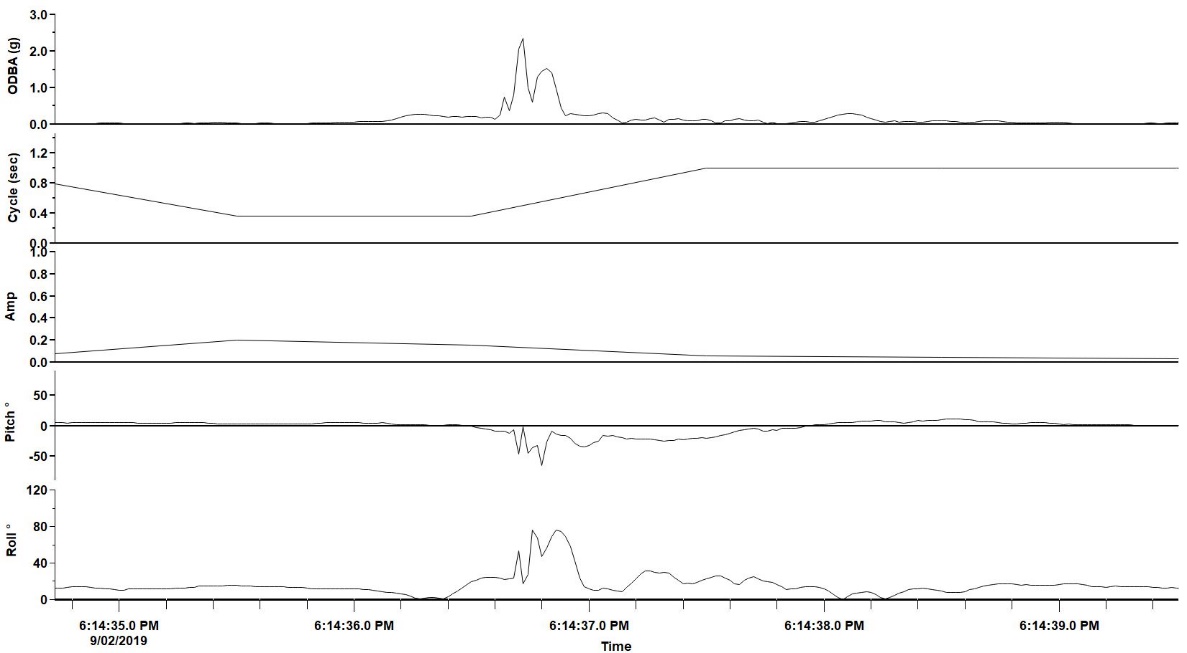


**C)**


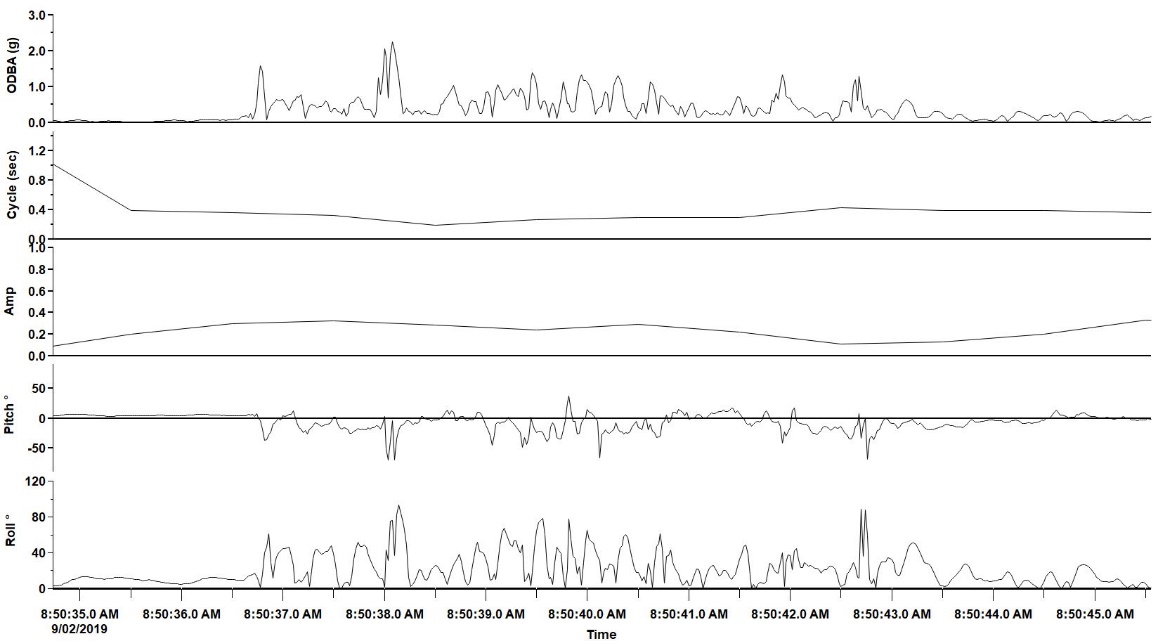


**D)**


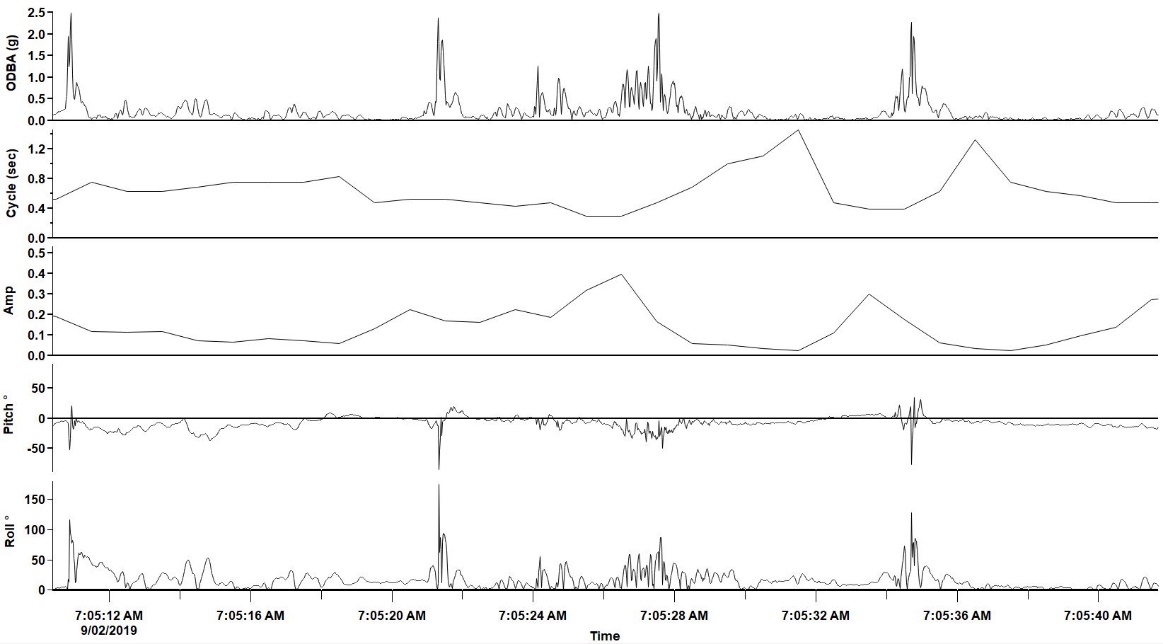


**E)**

***Figure S5.*** *Acceleration signatures for A) swim, B) escape, C) chafe, D) feed, and E) courtship behavioural classes from captive Yellowtail Kingfish recorded via accelerometer loggers.*


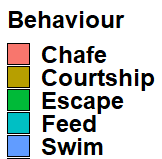

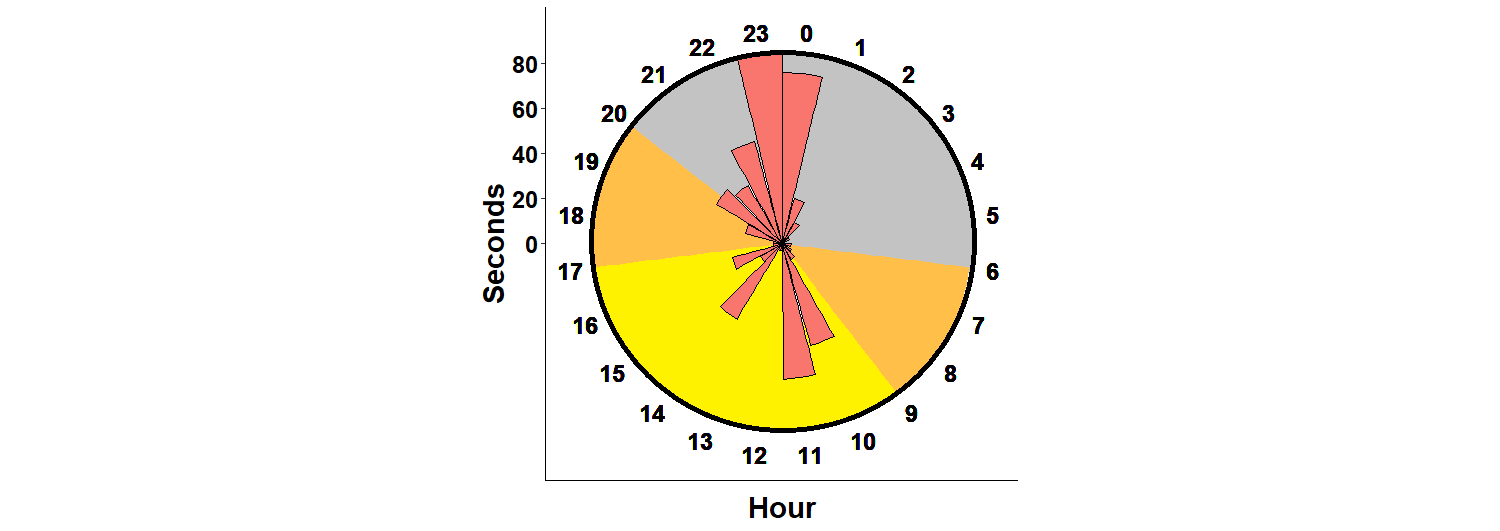

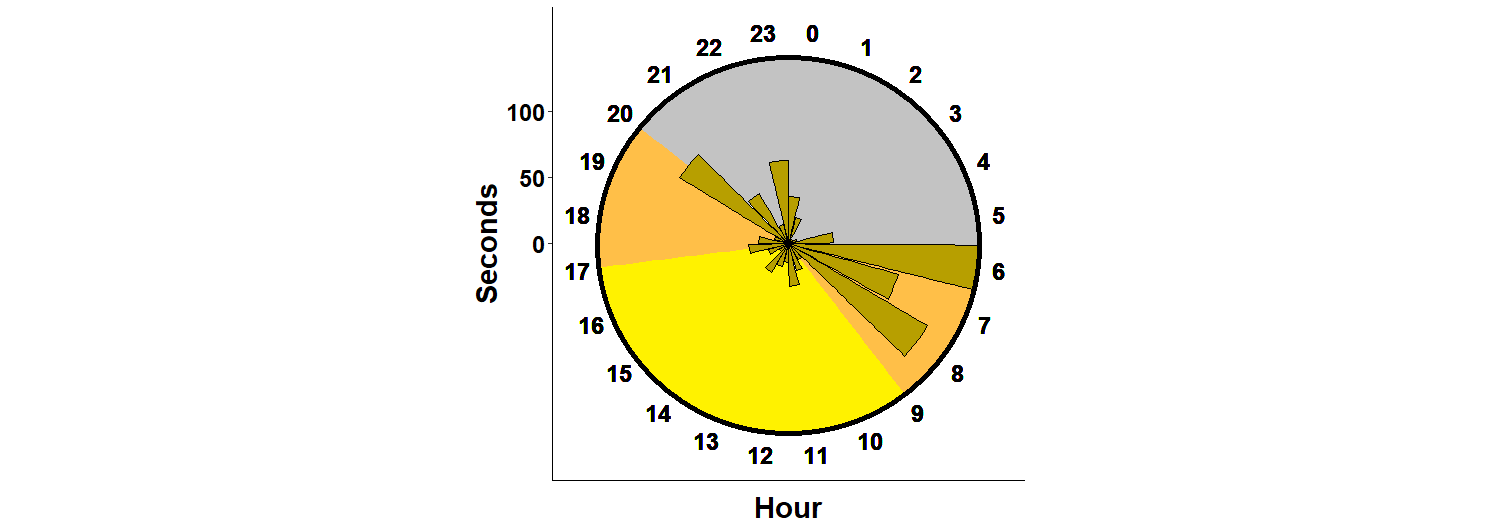

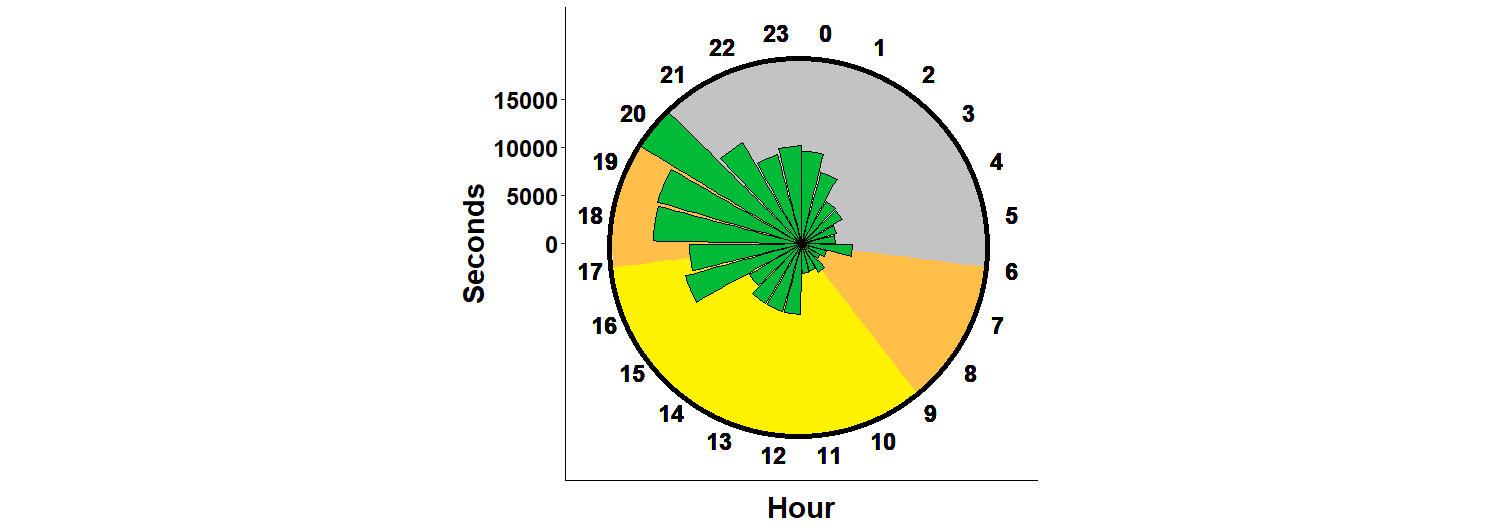

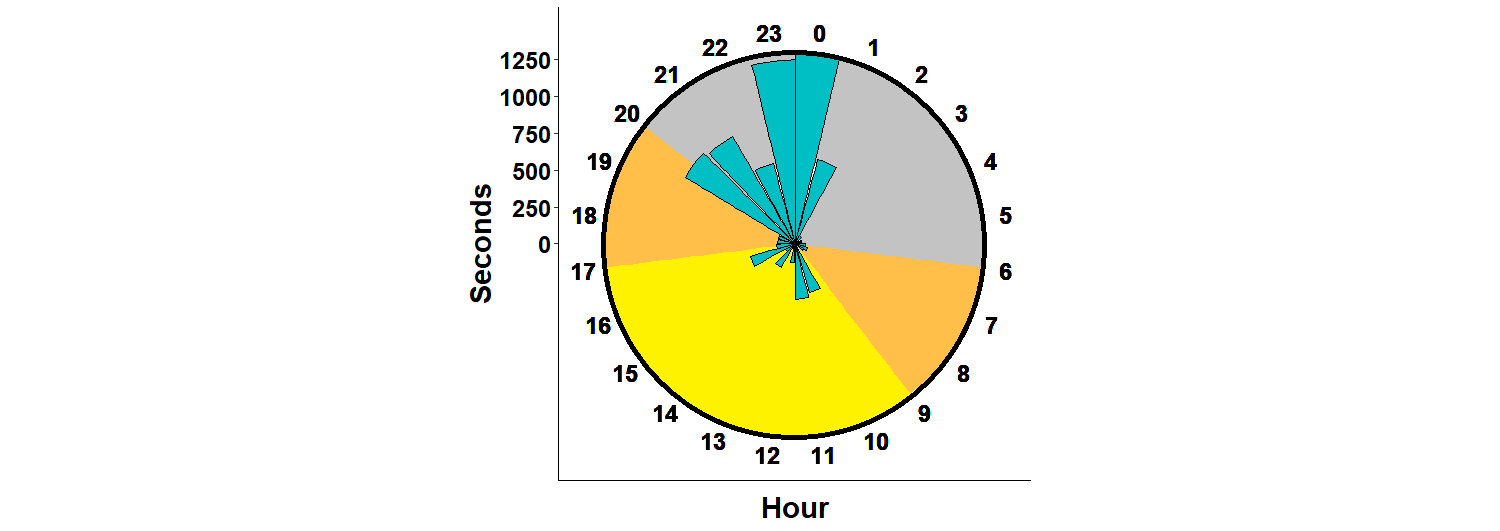

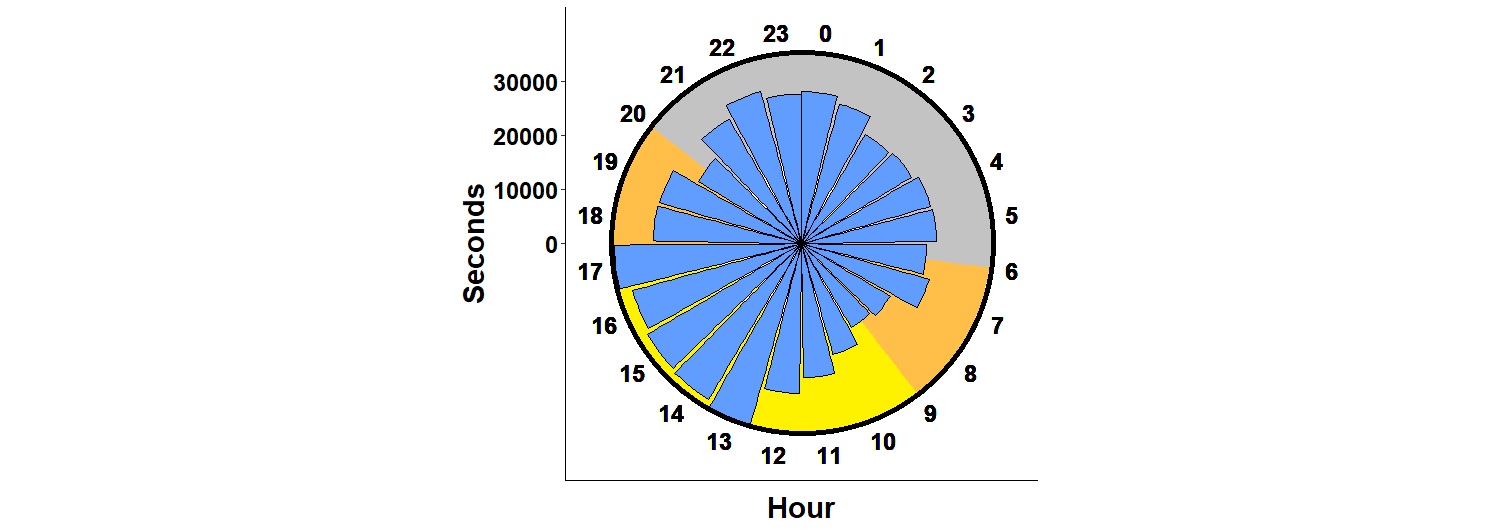


***Figure S6.*** *Number of 1 second increments predicted from free-ranging Yellowtail Kingfish at each hour of the day.* *Time of day is indicated by dawn (orange), dusk (orange), day (yellow) and night (grey).*


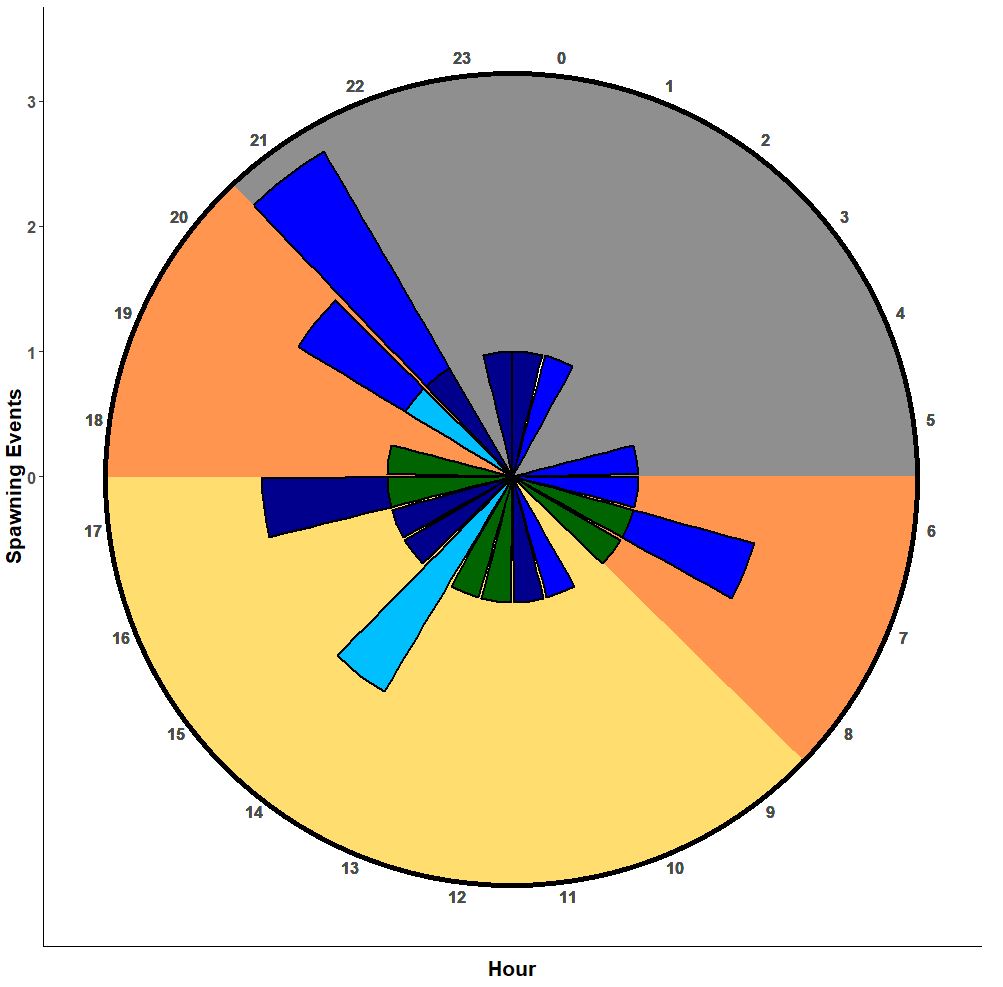

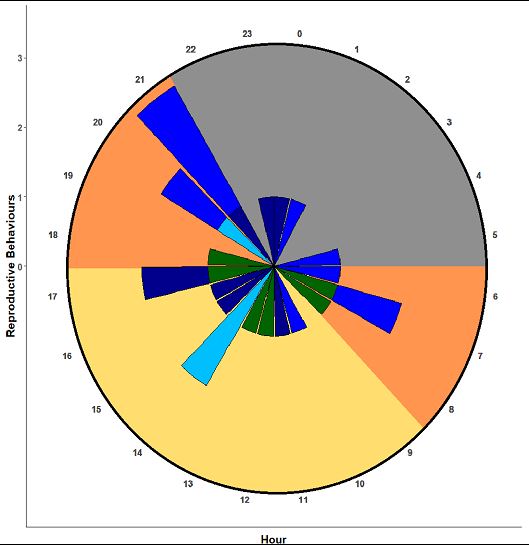


***Figure S7.*** *Number of a) reproductive behaviours and b) spawning events predicted from free-ranging Kingfish at the Neptune Islands (blue) and Coffin Bay (green), as predicted from the Random Forest model. Time of day is indicated by dawn (orange), dusk (orange), day (yellow) and night (grey).*

***Table S1****. Total number of seconds for each behaviour class from captive Yellowtail Kingfish used to train the Random Forest model*

| **Fish** | **Behaviour** | **Events** |
| --- | --- | --- |
| C1 | Chafe | 23 |
| C1 | Courtship | 164 |
| C1 | Escape | 2 |
| C1 | Feed | 30 |
| C2 | Chafe | 5 |
| C2 | Courtship | 0 |
| C2 | Escape | 0 |
| C2 | Feed | 169 |
| C3 | Chafe | 16 |
| C3 | Courtship | 172 |
| C3 | Escape | 2 |
| C3 | Feed | 0 |
| C4 | Chafe | 6 |
| C4 | Courtship | 128 |
| C4 | Escape | 134 |
| C4 | Feed | 274 |
| C5 | Chafe | 18 |
| C5 | Courtship | 153 |
| C5 | Escape | 78 |
| C5 | Feed | 245 |
| C6 | Chafe | 45 |
| C6 | Courtship | 149 |
| C6 | Escape | 182 |
| C6 | Feed | 614 |
